# Supplementary material for: Association of Vitamin D Deficiency and Insufficiency with Pathology in Hospitalized Patients
Source: Diagnostics (Basel). 2023 Mar 6;13(5):998. doi: 10.3390/diagnostics13050998 (PMC10000859; doi:10.3390/diagnostics13050998)
Supplement: Supplementary file 1 [file diagnostics-13-00998-s001.zip › diagnostics-2220510-supplementary.pdf]

Table S1 Correlation between sex, year, month, season and group ages with vitamin D status

			Vitamin D status		
	Factor		Deficiency	Insufficiency	Normal
Adjusted ratio, p<0,001	SEX	Males	7.3	0.6	-7.3
		Females	-7.3	-0.6	7.3
	YEAR	2020	-8.0	3.7	3.9
		2021	0.5	-1, 0.4	0.8
		2022	7.3	-2.0	-4.8
Month of the year	January		4.1	-2.9	-1.0
	February		5.0	-4,.6	-0.3
	March		6.1	-1.5	-4.2
	April		5.9	-.6	-4.9
	May		4.1	1.2	-5.0
	June		-1.1	2.5	-1.4
	July		-4.8	2.1	2.4
	August		5.7	1.5	3.9
	September		-7.5	1. .8	5.2
	October		-5.6	1.9	3.4
	November		-3.4	-.7	3.8
	December		0.1	-1.0	0.8
Season	Spring		10.6	-.6	-9.2
	Summer		-6.8	4.0	2.5
	Fall		-10.4	1.9	7.9
	Winter		6.0	-5.5	-0.3
Age	<20		2.7	-1.0	-1.5
	20-39		12.5	-4.9	-6.9
	40-59		-1.2	4.7	-3.3
	60-79		-11.7	3.4	7.5
	> 80		6.3	-4.6	-1.5

Table S2: The correlation with discharge first diagnostic;

		**Optimal (n=4367; 39.05%)**		**Insufficiency (n=3591; 32.11%)**		**Deficiency (n=3224; 28.83%)**	
	N (%)	p-value	OR, 95%CI	p-value	OR, 95%CI	p-value	OR, 95%CI
Infectious diseases	115 (1.0%)	<0.0001	OR=0,5, [0,337-0,741]	<0,01	OR=0,488, [0,298-0,799]	p<0,01, OR=3,762, [2,584-5,475	
Malignant neoplasm	118 (1.1%)	P<0,001		P=0.158		p<0,01, OR=2,673,[1,859-3845]	

Benign or uncertain behaviour neoplasms	199 (1.8%)	P=0,131	OR=0,501, [0,34-0,738]	P=0,615		P=0,66
Anaemias	80 (0.7%)	P=0,079		P=0,32		P=0,142
Diseases of blood and blood-forming organs	9 (0.08%)	P=0,807		P=0,76		P=0,661
Disorders of thyroid gland	2258 (20.2%)	P<0,001		P=0,001,	OR=1,183,[1,070-1,307]	P<0,001, OR=0,578,[0,517-0,646]
Diabetes mellitus	314 (2.8%)	P=0,76	OR=1,27, [1,158-1,393]	P=0877		P=0,484
Disorders of other endocrine glands and pancreas	83 (0.7%)	P=0,411		P=0,836		P=0,476
Malnutrition and nutritional deficiencies	193 (1.7%)	P=0,0355		P=0,17		P=0,62
Obesity and other hyperalimentation and metabolic disorders	579 (5.2%)	P=0,004		P=0,041	OR=1,205, [1,007-1,441]	P=0,447
Mental and behavioural disorders	507 (4.5%)	P=0,146	OR-0,779, [0,658-0,923]	P=0,816		P=0,17,OR=1,259, [1,042-1,521]
Diseases of the nervous system	407 (3.6%)	P=0,142		P=0,318		P=0,551
Diseases of the eye and adnexa	5 (0.04%)	P=0,159		P=0,666		P=0,155
Diseases of the ear and mastoid process	35 (0.3%)	P=0,494		P=0,693		P=0,434
Acute rheumatic fever and chronic rheumatic heart diseases	2 (0.02%)	P=0,966		P=0,369		P=0,509
Hypertensive diseases	17 (0.2%)	P=0,545		P=0,55		P,=0,629
Ischaemic heart diseases	135 (1.2%)	P=0,794		P=0,235		P=0,576
Other forms of heart disease and pulmonary heart diseases	816 (7.3%)	P<0,001,		P=0,027 OR=0,83		P<0,001, OR=1,714,[1,481-1,985]

				2,[0,707-0,98]		
Diseases of the respiratory system	1552 (13.9%)	P=0,009	OR=0,75, [0,649-0,868]	P=0,719		P<0,001, OR=1,26, [1,124-1,413]
Diseases of oral cavity, salivary glands and jaws	4 (0.04%)	P=0,289	OR=0,866, [0,776-0,964]	P=0,869		P=0,23
Diseases of oesophagus, stomach and duodenum	657 (5.9%)	P=0,205		P=0,914		P=0,447
Hernia and diseases of the appendix	21 (0.2%)	P=0,723		P=0,987		P=0,661
Noninfective enteritis and colitis	29 (0.3%)	P=0,253		P=0,493		P=0,501
Other diseases of intestines and diseases of peritoneum	68 (0.6%)	P=0,464		P=0,508		P=0,216
Liver, billiary and pancreatic diseases	922 (8.2%)	P=0,025	OR=1,167, [1,02-1,336]	P=0,13		P=0,294
Other digestive diseases	6 (0.05%)	P=0,118		P=0,514		P=0,41,
Diseases of the skin and subcutaneous tissue	33 (0.3%)	P=0,483		P=0,842		P=0,18
Diseases of the musculoskeletal system and connective tissue	1419 (12.7%)	P<0,001	OR=1,281, [1,146-1,432]	P=0,627		P<0,001, OR=0,71,[0,623-0,89]
Urinary and renal diseases	217 (1.9%)	P=0,001	OR=0,626, [0,475=-0,827]	P=0,117		P<0,001, OR=2,358, [1,801-3,088]
Unspecific	382 (3.4%)	P0,781		P=0,665		P=0,634
Cardiovascular disorders	4690	P<0,001	OR=0,742, [0,688-0,800]	P<0,001	OR=0,848,[0,78-0,921]	P<0,001,OR=1,785, [1,644-1,939]
Metabolic disorders	4296	P<0,001	OR=0,788, [0,73-0,85]	P=0,536		P<0,001, OR=1,409, [1,296-1,531]
Endocrine total	3736	P=0,643		P=0,674		P=0,785
Cancers total	343	P=0,001	OR=0,713, [0,584-0,869]	P=0,007	OR=0,727,[0,577-0,916]	P<0,001, OR=2,104, [1,728-2,562]

COVID-19	1913	P<0,001	OR=0,835, [0,757-0,922]	P=0,189		P<0,001, OR=1,381, [1,244-1,534]

Table S3: Correlation between comorbidities and vitamin D status.

**Adjusted ratio**	**Vitamin D status**					
	Factor	Deficiency	Insufficiency	Optimal	Pearson Chi-Square	P value
**Cardiovascular pathology**	positive	13,8	-4,0	-9,0	196,395	0.000
**Dysmetabolic status**	positive	8,1	0,3	-7,8	83,281a	0.000
**Endocrine disorders**	positive	-0,3	0,1	,2	0,080a	0,961
**Maglinancy**	positive	6,9	-3,8	-2,8	48,497a	0.000
**COVID-19**	positive	6,1	-1,3	-4,4	39,056a	0.000
